# Psychological interventions to prevent relapse in anxiety and depression: A systematic review and meta-analysis

**DOI:** 10.1371/journal.pone.0272200

**Published:** 2022-08-12

**Authors:** Esther Krijnen-de Bruin, Willemijn Scholten, Anna Muntingh, Otto Maarsingh, Berno van Meijel, Annemieke van Straten, Neeltje Batelaan

**Affiliations:** 1 Psychiatry, Amsterdam Public Health Research Institute, Vrije Universiteit, Amsterdam UMC, Amsterdam, The Netherlands; 2 GGZ inGeest Specialised Mental Health Care, Amsterdam, The Netherlands; 3 Health, Sports & Welfare, Cluster Nursing, Inholland University of Applied Sciences, Amsterdam, The Netherlands; 4 General Practice & Elderly Care Medicine, Amsterdam Public Health Research Institute, Vrije Universiteit, Amsterdam UMC, Amsterdam, The Netherlands; 5 Parnassia Psychiatric Institute, Parnassia Academy, The Hague, The Netherlands; 6 Clinical, Neuro and Developmental Psychology, Amsterdam Public Health Research Institute, Vrije Universiteit, Amsterdam UMC, Amsterdam, The Netherlands; Public Library of Science, UNITED KINGDOM

## Abstract

**Objectives:**

The aim of this review is to establish the effectiveness of psychological relapse prevention interventions, as stand-alone interventions and in combination with maintenance antidepressant treatment (M-ADM) or antidepressant medication (ADM) discontinuation for patients with remitted anxiety disorders or major depressive disorders (MDD).

**Methods:**

A systematic review and a meta-analysis were conducted. A literature search was conducted in PubMed, PsycINFO and Embase for randomised controlled trials (RCTs) comparing psychological relapse prevention interventions to treatment as usual (TAU), with the proportion of relapse/recurrence and/or time to relapse/recurrence as outcome measure.

**Results:**

Thirty-six RCTs were included. During a 24-month period, psychological interventions significantly reduced risk of relapse/recurrence for patients with remitted MDD (RR 0.76, 95% CI: 0.68–0.86, p<0.001). This effect persisted with longer follow-up periods, although these results were less robust. Also, psychological interventions combined with M-ADM significantly reduced relapse during a 24-month period (RR 0.76, 95% CI: 0.62–0.94, p = 0.010), but this effect was not significant for longer follow-up periods. No meta-analysis could be performed on relapse prevention in anxiety disorders, as only two studies focused on relapse prevention in anxiety disorders.

**Conclusions:**

In patients with remitted MDD, psychological relapse prevention interventions substantially reduce risk of relapse/recurrence. It is recommended to offer these interventions to remitted MDD patients. Studies on anxiety disorders are needed.

**Systematic review registration number:**

PROSPERO 2018: CRD42018103142.

## Introduction

Anxiety and depressive disorders are a major public health issue, affecting approximately 615 million people worldwide [1]. Comorbidity among these categories of disorders is high [2], transitions between anxiety and depression are frequent [3–5], these disorders impact upon each other [6–8], and both belong to the internalizing disorders sharing multiple etiological factors and psychopathological processes [9]. In recent decades, many treatments for anxiety and depression in the acute phase have proven effective [10, 11]. However, relapse is common in these patients [12, 13], even for those who have adequately responded to treatment in the acute phase. The term ‘relapse’ is used to refer to both relapse and recurrence, as these terms are often used interchangeably [14], indicating a return to full symptoms and meeting the criteria for anxiety disorder or MDD following remission or recovery [15]. Although a distinction between relapse and recurrence is described in the literature [15, 16], it appears most studies do not distinguish between the two [17]. Clarke et al. [18] suggest that this might be a result of a limited dissemination of these terms, although it might also be due to the fact that a distinction between the terms is not supported by evidence from intervention trials [19]. A large variation of relapse rates are reported, depending on definitions of relapse, populations, follow-up periods and the type of studies. In anxiety disorders, after remission, 14–58% of patients experienced a relapse [5, 8, 12, 20, 21], with similar relapse rates for subtypes of anxiety disorders [5]. Likewise, with regard to major depressive disorder (MDD), 18–77% of patients experienced a relapse [22–28].

Although characteristics such as having residual symptoms, prior episodes and childhood maltreatment [17] are known to increase relapse risks, mechanisms underlying relapse are still poorly understood. Most common explanations for the high relapse risk in anxiety disorders and MDD are based on two hypotheses: 1) some individuals have a greater premorbid vulnerability than others (for example due to childhood maltreatment), or b) the ‘scarring-hypothesis’, which suggests that each depressive episodes leaves residual effects that increase vulnerability for the next episode [29], caused by biological factors, cognitive factors and stress-related factors. Although some evidence based interventions seem to affect mechanisms underlying change and hence potentially change the risk of relapse [30], not all effective treatments in the acute phase guarantee a good prognosis over time in all patients. This suggests that relapse prevention might be beneficial for remitted patients to remain stable over time.

Guidelines for anxiety disorders and MDD generally recommend two strategies for preventing relapse after remission has been achieved: 1) continuation of antidepressant medication (ADM), and/or 2) psychological relapse prevention interventions [31–34]. Continuation of ADM after treatment in the acute phase reduces relapse rates [35–37]. Meta-analyses indicate that, when ADM is continued after the initial response to ADM, 16–18% of patients with remission from an anxiety or depressive disorder experienced relapse, while 36–41% of these patients relapsed if ADM was discontinued [35, 36].

However, psychological relapse prevention interventions might be a better treatment option for some patients than maintenance ADM (M-ADM) for several reasons. First, patients who experience serious adverse effects of M-ADM (e.g. sexual dysfunction, dry mouth) [38] might be reluctant to adhere to ADM during asymptomatic periods [39]. Indeed, non-adherence to ADM is common among remitted patients [40]. Second, patients might also prefer psychological interventions over M-ADM for relapse prevention. Although there are no studies to support this assumption in remitted patients, it is well known that patients engaging in acute treatment have a strong preference for psychological treatment over pharmacological treatment [41]. The frequent occurrence of discontinuation symptoms and relapse after discontinuation [42, 43] might play a part in this preference. This is in line with our clinical experience. A third reason, as reported in one meta-analysis, is that psychological interventions were more successful than ADM in preventing relapse, as patients receiving psychological interventions had 17% less risk of relapse than patients receiving M-ADM [44]. Therefore, psychological interventions are important in the prevention of relapse.

Meta-analyses focusing on psychological relapse prevention interventions for depressive disorders indicate that these interventions are effective in preventing relapse, with reductions of 22–50% in relapse [18, 44–48]. Most of the studies included focused on cognitive behavioural therapy (CBT), cognitive therapy (CT) and mindfulness-based cognitive therapy (MBCT). Meta-analyses conducted by Biesheuvel-Leliefeld et al. [44] and by Clarke et al. [18] also included studies on interpersonal therapy (IPT). To date, no systematic reviews and/or meta-analyses are available with regard to psychological interventions for preventing relapse in patients with remitted anxiety disorders. This is remarkable, given the high prevalence of anxiety disorders, particularly in light of the fact that relapse is prevalent in both anxiety disorders and MDD [8].

Previous meta-analyses contain very little information about the effectiveness of adding psychological interventions to M-ADM or the discontinuation of ADM, even though this approach could be promising for preventing relapse [49, 50]. Although meta-analyses have reported results of studies allowing the use of M-ADM, only one meta-analysis studied the effect of adding psychological interventions to ADM, and found that this significantly reduces relapse risks when compared to ADM only [50]. To our knowledge, there are no studies in depression directly comparing the addition of psychological interventions to discontinuation of ADM versus discontinuation alone. Furthermore, existing meta-analyses report on only a limited follow-up duration of 24 months, while studies with longer follow-up durations are becoming increasingly available. Moreover, no meta-analyses have been performed with regard to the effectiveness of psychological relapse prevention interventions for patients with remitted anxiety disorders. For clinical practice, it is also important to know whether the timing and type of interventions are associated with relapse risks. For example, it has not been consistently examined and reported whether relapse prevention interventions are more effective for patients who have received other interventions prior to the relapse prevention intervention [18, 44, 48]. Additional insight into influencing factors could provide recommendations for clinical practice.

The current systematic review and meta-analysis is intended to update current research, leading to more robust estimates of the effects of psychological interventions for preventing relapse. Besides, this study is intended to extend previous research by 1) including studies regarding the prevention of anxiety disorders, 2) including studies with longer follow-up durations, 3) studying the effects of adding psychological interventions to maintenance ADM or discontinuation of ADM, as most remitted patients are using ADM or discontinue their medication, and 4) performing subgroup analyses on timing and type of interventions. Furthermore, current gaps in research will be identified and these could serve as research agenda for future research. In short, the aim of this systematic review and meta-analysis is to examine the effectiveness of psychological relapse prevention interventions, as compared to treatment as usual (TAU), for patients with remitted anxiety disorders or MDD.

## Methods

### Design

To examine the effectiveness of psychological interventions, we conducted a systematic review and meta-analysis. We also performed subgroup-analyses and meta-regression analyses to investigate whether the timing and type of interventions were associated with risk of relapse. The study was conducted and reported according to the Preferred Reporting Items for Systematic Reviews and Meta-Analyses (PRISMA) statement [51]. The protocol for this systematic review and meta-analysis was registered in PROSPERO with the number CRD42018103142 (https://www.crd.york.ac.uk/prospero/display_record.php?ID=CRD42018103142).

### Literature search

We searched PubMed, PsycINFO and Embase (from inception to July 2021) for randomised controlled trials (RCTs) including patients with a remitted anxiety disorder or MDD who had received a psychological intervention to prevent relapse, comparing this intervention to TAU, and reporting on relapse rate and/or time to relapse. A certified librarian (CP) and EKB performed the search using the following search terms: depressive disorder, anxiety disorder, psychotherapy, relapse/recurrence, and randomised controlled trials (RCTs). Terms were adapted for each database, and no limits or filters were applied (see S1 File). Only published articles written in English or Dutch were included. In addition, we searched reference lists of relevant articles for additional studies.

The following inclusion criteria were applied: a) a randomised controlled trial (RCT), b) examining adult patients (18 years and older) with a prior anxiety disorder and/or MDD, c) who were in remission at randomisation, d) receiving a psychological intervention with the aim of preventing relapse, e) compared with TAU, and f) with relapse rates and/or time to relapse as outcome.

For ‘remission’, ‘relapse’ and ‘recurrence’, we used the definitions applied in the original articles. No time limits were applied with regard to when patients had experienced their prior anxiety disorders and/or MDD.

All follow-up durations were allowed. We considered psychological relapse prevention interventions as stand-alone treatments, as well as psychological relapse prevention interventions combined with M-ADM or with discontinuation of ADM. Psychological relapse prevention interventions as stand-alone treatments were compared to TAU. TAU was considered as treatment that patients would normally receive, and could consist of no treatment at all; evaluation only; monitoring; non-specific support; or any other treatment that was not specifically aimed at relapse prevention. Therefore, studies in which two psychological interventions aimed at preventing relapse were compared to each other were excluded. Because maintenance treatment with antidepressants in itself reduces the risk of relapse, and because discontinuation of antidepressants in itself increases such risks, studies in which a psychological relapse prevention intervention was given in combination with one of these treatment strategies were considered separately. When examining the effect of psychological relapse prevention interventions in combination with M-ADM, the control group also consisted of M-ADM. Likewise, when psychological prevention interventions were given in combination with discontinuation of ADM, the control group also consisted of discontinuation of ADM. An overview of interventions and controls is provided in Table 1. Stepped care studies were excluded, because not all patients in one condition received the same treatment.

**Table 1:** Overview of comparisons of interventions and control groups, for anxiety and depression studies with different follow-up durations.

Intervention	Control
Psychological interventions	Treatment as usual
Psychological interventions + M-ADM	M-ADM
Psychological interventions + discontinuation of ADM	Treatment as usual + discontinuation of ADM

Note: psychological interventions = cognitive behavioural therapy (CBT), cognitive therapy (CT), preventive cognitive therapy (PCT), internet-based CBT, continuation cognitive therapy (C-CT), maintenance cognitive behavioural therapy (M-CBT), mobile cognitive therapy, mindfulness-based cognitive therapy (MBCT), interpersonal psychotherapy (IPT), (cognitive) psychoeducation ((C)PE) with therapeutic components, cognitive-behavioural analysis system of psychotherapy (CBASP); M-ADM = maintenance antidepressant medication; treatment as usual = no treatment, evaluation only, monitoring, non-specific support.

The screening of titles and abstracts was performed by three researchers: EKB screened all of the records, with WS and JG each screening half of the records. The computer programme ‘Rayyan’ was used to facilitate this process [52]. After the initial screening, the full-text screening was also performed by three researchers: EKB screened all of the records, with WS and JG each assessing half of the records. Few disagreements occurred and these were resolved by consensus-based discussion until consensus was reached.

### Data extraction

Data were extracted using a template based on the Cochrane Data Extraction and Assessment Template [53]. The following data were extracted: 1) participant characteristics (including age, gender, number of previous episodes required for inclusion in the study, number of participants), 2) study characteristics (including study setting, definition of remission, definition of relapse, relapse rates, duration of follow-up) and 3) intervention and comparison characteristics (including type and duration of intervention). Each of the three researchers independently extracted the data from the articles using this template. These sheets were subsequently compared to check the extracted data. In the event of uncertainties about the data, the authors of the original articles were contacted to provide clarification. Remaining disagreements were resolved by discussion with NB until consensus was reached. When relapse rates were not provided in the article, they were computed based on the number of relapses and the total number of patients in each group.

### Quality assessment

We used the Cochrane Collaboration’s tool for assessing risk of bias in order to assess the quality of the studies [54]. For each study, the risk of bias was assessed for the domains ‘random sequence generation’, ‘allocation concealment’, ‘blinding of outcome assessment’, ‘incomplete outcome data’, ‘selective reporting’ and ‘other bias’, with a low, high or unclear risk. This task was performed by two researchers (EKB and WS). Disagreements were resolved by discussion until consensus was reached.

### Meta-analysis

We aimed to establish the effectiveness of psychological relapse prevention interventions in anxiety disorders and MDD, planning separate analyses to synthesise studies including patients with anxiety disorders and MDD and distinguishing three intervention groups—psychological interventions a) as stand-alone treatments, b) in combination with M-ADM and c) in combination with ADM discontinuation—along with their control counterparts (i.e. TAU, M-ADM, discontinuation of ADM). The primary outcome measures planned were proportion of relapse and time to relapse. Separate analyses were planned for studies with a follow-up period of <24 months and >24 months. When multiple follow-up points were available <24 months, the follow-up point closest to 24 months was selected. In studies with a follow-up >24, the longest available follow-up point was chosen. Meta-analysis was used to synthesise the findings. In cases where there was a paucity of data, no meta-analysis could be performed. This was the case for anxiety disorders as only two studies were found regarding the effectiveness of psychological interventions for the prevention of relapse of anxiety disorders [55, 56], and for studies in which ADM was discontinued, that additionally had different follow-up durations. For anxiety disorders and studies in which ADM was discontinued, qualitative description of the data was provided instead. Furthermore, time to relapse was often not reported. Even when it was reported, there was too much variation in the presentation of the results to allow for summarisation. The effect of interventions on time to relapse was therefore not analysed.

Meta-analysis measured effect sizes measured as risk ratios for relapse. Risk ratios with 95% confidence intervals were chosen, as they are more conservative than odds ratios are [57], and they can be easily compared with other systematic reviews [18, 44–46]. Effect sizes were based on intention-to-treat (ITT) data. A p value of < 0.05 was considered as statistically significant. The studies included differed according to various aspects, including type of disorder, interventions and demographic variables. Heterogeneity was therefore assumed. For this reason, a random effects model was used for the meta-analysis of the studies. Heterogeneity was explored using the Q-value and the I^2^ statistic. A significant Q-value (p < 0.05) indicates evidence of heterogeneity [58]. The I^2^ statistic is the ‘percentage of total variation across studies that is due to heterogeneity rather than chance’. It can range from 0% to 100%, and can roughly be interpreted as follows: 0–40% might not be of importance, 30–60% may be considered as moderate heterogeneity, 50–90% may represent substantial heterogeneity and >75% may reflect considerable heterogeneity [59].

Separate analyses were planned to synthesise studies including patients with anxiety disorders and MDD. Studies that allowed medication use were included in the main analysis, and the impact of including these studies was assessed using a sensitivity analysis. Studies in which medication use was not allowed were subjected to separate meta-analysis, and these results were compared to those of the main analysis.

Studies with two intervention groups and two control groups were analysed separately. For studies with two eligible interventions and one control group, the control group was split in half for the purpose of analysis, as proposed by Higgins et al. [59].

When possible, subgroup analyses were a priori defined and performed with regard to whether patients had received any intervention (psychological or pharmacological) prior to the relapse prevention intervention (yes/no), type of intervention (e.g. MBCT and CBT), setting (community, primary care, specialised care) and mode of delivery (online/face-to-face, guided/unguided). Meta-regression analyses were also a priori defined and performed to estimate the influence of the number of earlier episodes required for inclusion in the study and the duration of the interventions (in weeks) on the outcomes of the study. All subgroup analyses and meta-regression analyses were performed on the studies included in the main analysis.

The software package Comprehensive Meta-Analysis version 3.0 was used for analyses [60].

## Results

**Fig 1:**
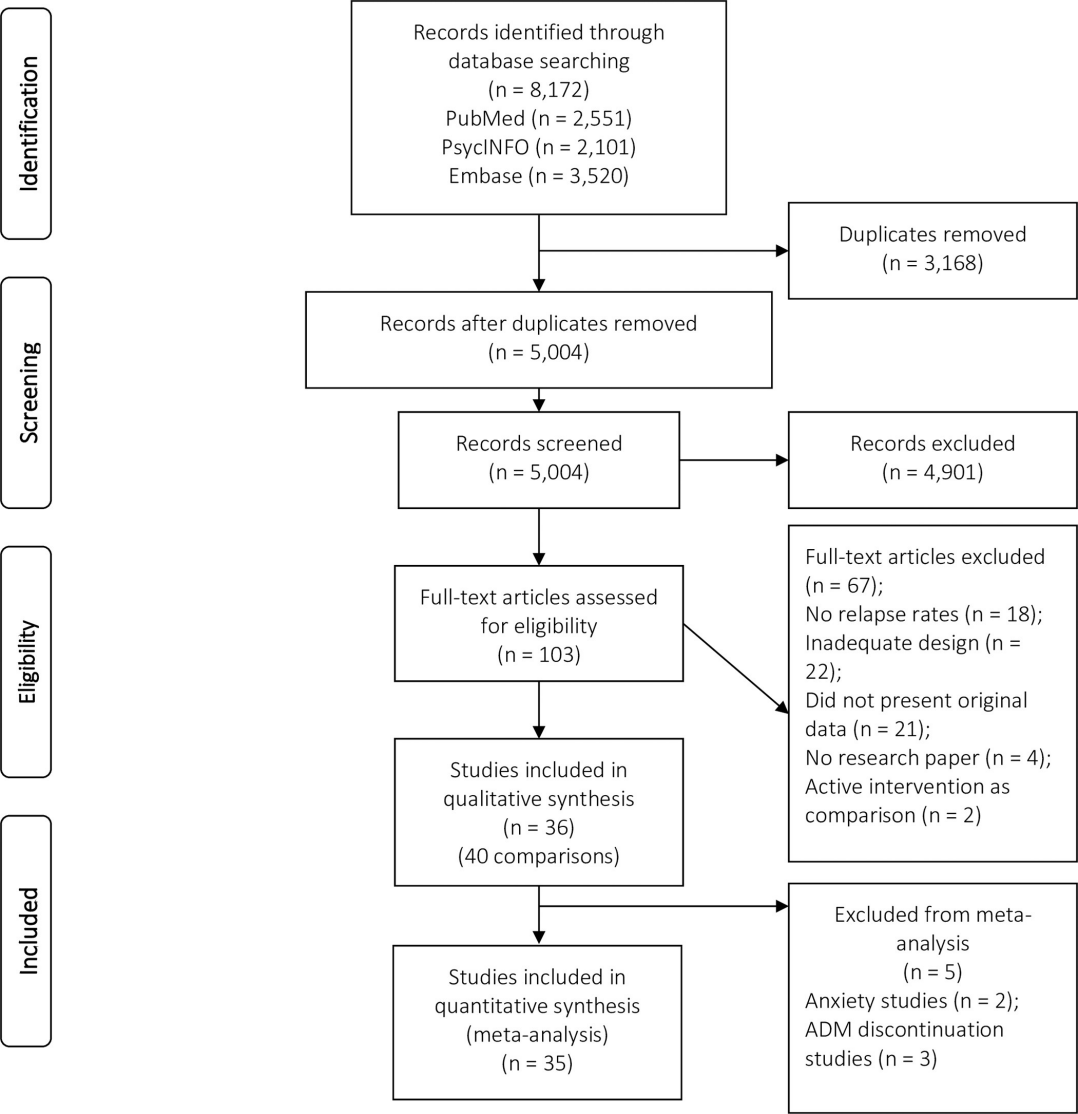
PRISMA flow diagram of the studies included.

The titles and abstracts of 5,004 records were screened, after removing 3,168 duplicates. Of these records, 103 were assessed as full-text, 36 studies (40 comparisons) were included in the qualitative synthesis and 35 comparisons were included in the quantitative synthesis (Fig 1). No additional studies were found by searching the reference lists of relevant articles. Of the 40 included comparisons, two (5%) comparisons were found regarding the effectiveness of psychological interventions for the prevention of relapse of anxiety disorders, 21 (53%) comparisons regarding the psychological interventions for depression with a maximum follow-up of 24 months, and 5 (13%) on depression with a follow-up > 24 months. Further, 6 (15%) comparisons were found on the effectiveness of psychological interventions and M-ADM < 24 months, and 3 (8%) comparisons with > 24 months follow-up). Three comparisons (8%) examined psychological relapse prevention plus antidepressant discontinuation versus TAU plus antidepressant discontinuation. Given that different studies had different durations of follow-up, we conducted separate analyses of studies with a follow-up duration up to and including 24 months and those with a follow-up duration of more than 24 months. This made it possible to include multiple papers about the same study with different follow-up durations (e.g. one up to and including 24 months and one of more than 24 months). For the main analysis, a follow-up duration up to and including 24 months was chosen with TAU as the control group.

### Characteristics of the studies

The characteristics of the studies are presented in S2 File. The studies were published between 1990 and 2020. Sample sizes for the 36 studies (40 comparisons) that were included in the meta-analyses ranged from 14 to 460. In all, the studies concerned 3,729 unique patients, with 1,949 in the intervention groups and 1,780 in the control groups. Sixteen of the studies were conducted in specialised care. Seventeen studies evaluated some variant of cognitive behavioural therapy, according to the description of the original articles: cognitive behavioural therapy (CBT) (number of studies [*k*] = 4), cognitive therapy (CT) (*k* = 4), preventive cognitive therapy (PCT) (*k* = 3), internet-based CBT (*k* = 2), continuation cognitive therapy (C-CT) (*k* = 2), maintenance cognitive behavioural therapy (M-CBT) (*k* = 1) and mobile cognitive therapy (*k* = 1). Eleven trials evaluated mindfulness-based cognitive therapy (MBCT), five trials evaluated interpersonal psychotherapy (IPT), two trials evaluated (cognitive) psychoeducation ((C)PE) with therapeutic components and one trial evaluated cognitive-behavioural analysis system of psychotherapy (CBASP). The duration of the interventions ranged from 6 to 156 weeks, and the duration of follow-up ranged from 6 to 66 months. Sixteen comparisons offered face-to-face contacts in group format, 23 in individual format (of which 5 internet-based or self-help) and 1 individual or group. Almost all (35) interventions were standardized, followed a strict protocol, and most studies offered around 10 sessions. The mean number of episodes required for study entry was 2. In addition, most of the studies included in our meta-analysis had a follow-up duration up to and including 24 months (*k* = 27).

Three studies [61–63] had two intervention groups and two control groups. One study had two intervention groups and one control group [64]. The total number of comparisons is therefore 26 for stand-alone psychological interventions and 9 for M-ADM combined with a psychological relapse prevention intervention, as compared to M-ADM only. Several studies had multiple follow-up points, varying from 26 to 66 months [65–72].

### Risk of bias appraisal

The risk-of-bias assessment for each study is summarised in S3 File. Risk of bias was generally low on the domains ‘random sequence generation’ (68% low risk of bias), ‘allocation concealment’ (58% low risk of bias), ‘blinding of outcome assessment’ (70% low risk of bias) and ‘incomplete outcome data’ (60% low risk of bias). As it was not possible to conceal psychological interventions from participants and personnel, all studies had a high risk of performance bias, and this is therefore not reported in S3 File. Most studies had an unclear risk of selective reporting bias, as many studies were not registered, study protocols were missing or pre-specified primary outcome measures were not reported. The risk of other bias was heterogeneous: 17 studies had a low risk of other bias, 9 had a high risk (e.g. due to specific problems mentioned by the authors, flaws in design, the absence of structured interviews or small sample) and 10 studies had an unclear risk of other bias.

### Studies on anxiety disorders

Our literature search revealed two papers on the prevention of relapse in patients with remitted anxiety disorders. White et al. [56] compared M-CBT to assessment only, and included only patients for whom anxiolytic medication had already been discontinued. Patients in the M-CBT group had significantly lower relapse rates (5.2%) compared to those in the assessment-only group (18.4%) at 21-month follow-up.

Scholten et al. [55] compared a CBT intervention plus discontinuation of ADM with discontinuation of ADM alone. They found no significant difference in relapse rates between patients in the intervention group and those in the control group (67% vs. 65%).

### Studies on depressive disorders

#### Psychological interventions versus treatment as usual

**Fig 2:**
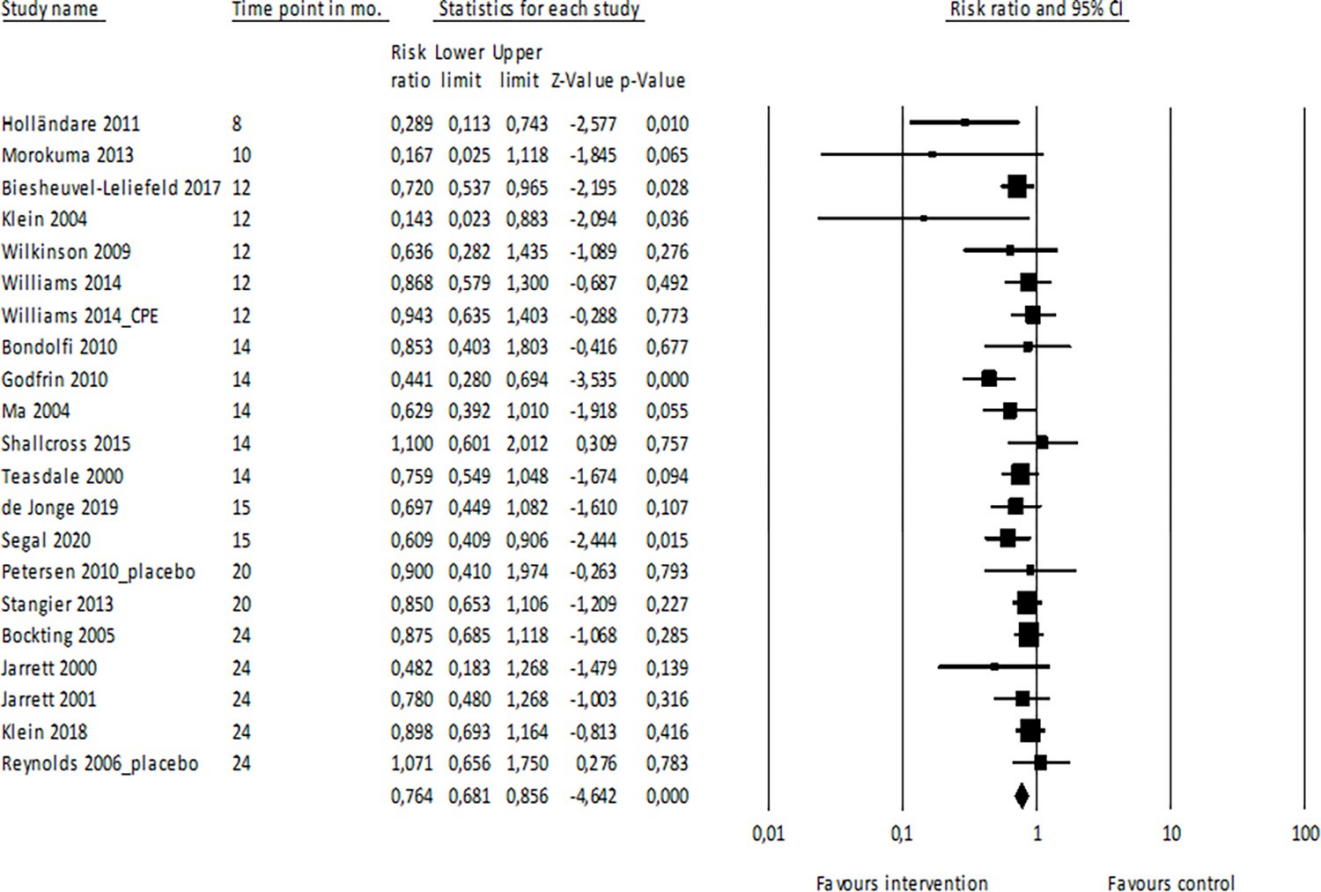
Meta-analysis of psychological interventions vs. TAU, up to and including 24 months. Note. CI = confidence interval; CPE = cognitive psychoeducation.

*Main analysis*: *≤24 months*. Data were available for 21 comparisons with a follow-up duration up to and including 24 months (Fig 2). These studies had an average follow-up duration of 16.2 months, with a range of 8 to 24 months. In all, 2,715 patients were included in this analysis. The summary risk ratio of relapse was 0.76 (95% CI: 0.68–0.86, p<0.001, I^2^ = 25.312) for patients in the psychological intervention group versus TAU, indicating that risk of relapse was reduced by 24% for patients who received psychological relapse prevention interventions, as compared to those who received TAU. For patients in the intervention group, the summary relapse prevalence was 34.7% (95% CI: 28.0% - 41.9%), while for patients in the TAU group the summary relapse prevalence was 47.2% (95% CI: 40.0% - 54.6%). The Duval and Tweedie trim-and-fill procedure indicated a slight change in the RR after adjustment (summary adjusted risk ratio 0.80, 95% CI: 0.70–0.92), with four imputed studies.

Several of the studies (n = 15) included in this meta-analysis allowed the use of ADM during the intervention [61, 62, 64, 65, 67, 71, 73–81], while other studies did not [82–86]. A sensitivity analysis with only studies in which medication was not allowed (number of comparisons = 5) revealed a risk ratio of 0.67 (95% CI: 0.50–0.90, p = 0.007). These results did not significantly differ from studies in which medication use was allowed.

Subgroup analyses. Differences in whether patients had received an intervention (psychological or pharmacological) prior to the relapse prevention intervention (yes/no) and type of intervention (CBT/MBCT) did not significantly affect the risk of relapse. IPT was not included in the subgroup analysis for type of intervention, as only one study was available with results up to and including 24 months [62]. Of note, this study showed no significant difference between the intervention and the control group. Planned subgroup analyses on setting and mode of delivery could not be performed, as there was either not enough or too much variation in the data. The subgroup analyses can be found in S4 File.

Meta-regression analyses. There was no statistically significant relationship between the number of previous episodes required for inclusion in the study and the outcome (B = 0.02, 95% CI: -0.14 to 0.19, p = 0.77). The influence of the duration of interventions was also not related to the outcome (B = 0.003, 95% CI: -0.003 to 0.007, p = 0.34).

*>24 months*. Five studies were included in the meta-analysis for studies with a follow-up duration of more than 24 months, with a total of 588 patients. Three studies in this meta-analysis were follow-ups to studies that were included in the main analysis [66, 68, 72]. The average follow-up period in this group was more than 3 years (38.6 months, range 26 to 66 months). The risk ratio of relapse in these studies was 0.78 (95% CI: 0.62–0.98, p = 0.036) for patients in the psychological intervention group versus TAU, indicating that risk of relapse was reduced by 22% for patients who received psychological relapse prevention interventions, as compared to those who received TAU. For patients in the intervention group, the summary relapse prevalence was 49.6% (95% CI: 29.6% - 69.8%), while for patients in the TAU group the summary relapse prevalence was 70.0% (95% CI: 51.8% - 83.6%). This effect is similar to the effect up to and including 24 months, but with greater heterogeneity (Q-value = 10.325, df = 4, p = 0.035, I^2^ = 61.261). These findings therefore suggest that the preventive effect persists over time.

#### Psychological interventions plus M-ADM versus M-ADM

*≤24 months*. In the meta-analysis of studies with a follow-up duration up to and including 24 months, which compared psychological relapse prevention interventions plus M-ADM to M-ADM only, six studies were included, with a total of 651 patients. The average follow-up in this group was 17.3 months, with a range of 6 to 24 months. When comparing psychological interventions plus M-ADM with M-ADM only, the risk ratio of relapse for these studies is 0.76 (95% CI: 0.62–0.94, p = 0.010, I^2^<0.001), indicating that risk of relapse was reduced by 24% for patients who received psychological relapse prevention interventions, as compared to those who received TAU. For patients in the intervention plus M-ADM group, the summary relapse prevalence was 26.9% (95% CI: 16.4% - 40.8%), while for patients in the M-ADM only group the summary relapse prevalence was 31.9% (95% CI: 18.5% - 49.2%). The addition of psychological interventions to M-ADM appears to be effective in preventing relapse.

*>24 months*. Three studies with a follow-up period of more than 24 months compared psychological interventions plus M-ADM to M-ADM only, with a total of 264 patients. Of these three studies, one was a follow-up to another study that was included in the meta-analysis up to and including 24 months [70]. The average follow-up in this group was more than 4 years (52.2 months, range of 36 to 63 months). The risk ratio of relapse for these studies is 0.87 (95% CI: 0.62–1.21, p = 0.396), indicating a reduction of 13% for patients receiving psychological relapse prevention. For patients in the intervention plus M-ADM group, the summary relapse prevalence was 34.2% (95% CI: 12.6% - 65.1%), while for patients in the M-ADM only group the summary relapse prevalence was 43.4% (95% CI: 20.2% - 69.9%). The addition of psychological interventions to M-ADM does not appear to be significantly effective in preventing relapse over a follow-up period of more than 24 months. Limited heterogeneity was found (Q-value = 2.357, df = 2, p = 0.308, I^2^ = 15.158).

#### Psychological interventions plus discontinuation of ADM vs. TAU plus discontinuation of ADM

Three studies were found in which ADM was discontinued in the intervention group, as well as in the TAU group [87–89]. As there were only three studies with different follow-up periods, we did not conduct a meta-analysis of these studies. Fava et al. [87] and Fava et al. [88] both had a follow-up period of 29 months and Segal et al. [89] had a follow up of 18 months. In one study, the relapse rate was significantly lower in the discontinuation plus CBT group (25%), as compared to the discontinuation group (80%) [88]. The relapse prevention effect was not significant in the other two studies [87, 89]. Based on the relapse rates found in the three individual studies, patients in the intervention plus discontinuation group showed relapse rates from 15% to 38%, while for patients in the TAU plus discontinuation group the relapse rates ranged from 35% to 80% (see S2 File).

## Discussion

### Findings and comparison with existing literature

This study focuses on psychological relapse prevention interventions for patients with remitted anxiety disorders or MDD. Due to a lack of studies on anxiety disorders (N = 2), no meta-analysis was performed with regard to the effectiveness of psychological relapse prevention interventions for patients with remitted anxiety disorders. Moreover, the two studies had contradictory findings so no clear conclusion could be drawn with regard to the effectiveness of relapse prevention interventions for patients with anxiety disorders. For patients with remitted MDD, psychological interventions reduced the risk of relapse by 24% on average, as compared to TAU within the first 24 months after the start of a relapse prevention intervention. Evidence suggested that this effect persisted for up to 3 years. When psychological interventions were offered in combination with M-ADM, the risk of relapse was also reduced by 24% on average compared to M-ADM alone within the first 24 months, although this did not remain significant over a longer period. Due to a paucity of studies, no meta-analysis was performed with regard to the effectiveness of psychological relapse prevention interventions combined with discontinuation of ADM. However, the three included studies all reported a better outcome (of which one reported a statistically significant difference) for patients receiving a psychological prevention intervention combined with discontinuation of ADM compared to patients receiving TAU with discontinuation of ADM.

Our results are consistent with those on relapse prevention in depressive disorders as reported by Clarke et al. [18] as they showed a 22% reduction in relapse rate, and lower than the 36% reduction that was found on relapse prevention in depressive disorders by Biesheuvel-Leliefeld et al. [44], when preventive psychological interventions were compared with TAU. The latter difference might be explained by the fact that inclusion criteria in our study were more strict regarding remission at randomisation. Therefore, we excluded a number of studies, which were included by them [90–95]. As they included studies with patients who had more severe symptoms, these patients might have experienced more benefit from the relapse prevention interventions. This could explain the larger relapse rate reduction found by Biesheuvel-Leliefeld et al. [44]. In contrast to these studies, we included more comparisons in our meta-analysis, as the process of splitting follow-up durations into two categories (up to and including 24 months and more than 24 months) allowed us to include multiple follow-up periods for the same study. This is in contrast to the analytical strategy applied by Clarke et al. [18] and by Biesheuvel-Leliefeld et al. [44], who considered only the results of one follow-up period for each study. In addition, we expanded their meta-analyses to include more recent studies, as these two meta-analyses included studies until 2013 and 2014, respectively. Both the inclusion of multiple follow-up periods for the same study and the addition of recent studies may result in a more reliable effect size compared to these earlier meta-analyses.

Although we might assume that the effect of psychological interventions decreases as follow-up time increases, the studies with a mean follow-up duration of over 3 years indicated that psychological interventions still appear to protect against relapse when compared to TAU. However, these findings were less robust than the findings up to and including 2 years. This study is the first to report meta-analytical results over a follow-up period of more than 2 years.

This study also compared psychological relapse prevention interventions plus M-ADM to M-ADM only. Psychological interventions were effective in preventing relapse up to and including 2 years, but no positive effect could be established after a longer follow-up period of more than 2 years. This could have been due to limited power in the meta-analysis, as only three studies were included. Other meta-analyses mostly compared psychological interventions to ADM [44, 46, 48], and only one meta-analysis studied the additional effect of psychological interventions [50]. As many remitted patients use M-ADM, and M-ADM in itself affects relapse rates [36], this comparison is highly relevant for clinical practice.

In this study, the effect sizes of the various types of interventions were comparable. This finding was also reported by Biesheuvel-Leliefeld et al. [44]. We found that CBT was effective in preventing relapse, as also reported by Zhang et al. [48] and by Clarke et al. [18]. In addition, we found that MBCT was effective in preventing relapse, as previously reported by Piet and Hougaard [46], Clarke et al. [18], and Zhang et al. [48]. Theoretically, preventive CT targets the content of cognition as key mechanisms for relapse. Dysfunctional beliefs are assumed to be latent in the remitted phase, but can be triggered by life events, stress or sad mood, and thereby cause recurrence of depression. MBCT on the other hand, is presumed to target both the process, as well as the content of cognition. It is supposed to help develop a detached and decentred relationship to thoughts and feelings, breaking the connection between mood reactivity and recurrence of depression. However, few studies have directly tested mediation of preventive psychological interventions in relapse and recurrence prevention. Further research is required to understand the working mechanism of psychological relapse prevention interventions [96].

The results of this study also appear to extend previous observations. As suggested by Zhang et al. [48], we analysed whether the effectiveness of psychological interventions differed for patients who had received an intervention (psychological or pharmacological) prior to the relapse prevention intervention, and we found no differences between the two subgroups. This is in contrast to the study by Biesheuvel-Leliefeld et al. [44], which found that psychological relapse prevention interventions were more effective if they were offered shortly after the conclusion of active treatment.

Another important finding of our analysis was that studies on psychological relapse prevention in anxiety disorders are scarce. Only two studies on this topic could be included in our systematic review and, for this reason, no meta-analysis was conducted. These studies differed substantially as Scholten et al. [55], who reported a negative result, discontinued ADM during the study, whereas White et al. [56] reported a positive result by providing psychological relapse prevention to patients who had already discontinued ADM before randomisation. It is possible that differences in patient population account for these discrepancies.

The scarcity of studies on anxiety disorders was surprising, given that relapse is prevalent in both anxiety disorders and in depressive disorders [8]. One explanation might be the prevailing idea among professionals that treatment for anxiety disorders is more effective in the long-term than treatment for depressive disorders, along with a possible lack of awareness regarding the unfavourable long-term course of anxiety. Professionals might therefore think that relapse prevention is less necessary for this group of patients.

### Strengths and limitations

This study has several strengths. Firstly, it is the first study to analyse data over a follow-up period of 3 years. Secondly, in contrast to other meta-analyses [18, 44, 48], we included more recent trials in our analyses, addressing not only patients suffering from MDD but also from anxiety disorders. A third strength of this study is the inclusion of different treatment strategies (and related control groups), which enabled us to analyse both the stand-alone effect of psychological interventions and their add-on effect to M-ADM.

This study is also subject to several limitations. Firstly, it was not possible to execute some of the planned analyses, due to incomplete data (e.g. on the effectiveness of psychological interventions for anxiety disorders and in combination with discontinuation of ADM). Secondly, differences in methodological designs were found in the studies included (e.g. the definition of relapse or remission and the different measures used to assess relapse and remission). This might have resulted in heterogeneity in the data. Thirdly, evidence of publication bias was found, although this did not significantly change the estimated effect size. Fourth, with the current study we could not establish whether the effect found was due to the fact that patients had more therapy sessions while receiving psychological relapse prevention interventions (compared to receiving no therapy sessions at all), or due to the content of the interventions themselves.

### Implications for future practice and research

Given that psychological relapse prevention interventions for remitted depressed patients reduce the risk of relapse by 24% on average, relapse prevention should be considered for all patients who are in remission from MDD, at least in those at high risk for relapse, as these patients may benefit most from relapse prevention interventions [17]. This corresponds to current guideline recommendations about providing psychological relapse prevention interventions after remission had been achieved [31–34]. Moreover, as our results suggest an additive effect of psychological relapse prevention on M-ADM up to and including 2 years, psychological relapse prevention should be considered for all patients on M-ADM in the 2 years after remission.

This study highlighted several gaps in current knowledge, and provides input for the research agenda in this field. First, it reveals a need for research into relapse prevention for patients with anxiety disorders, as this could provide insight into the effectiveness of psychological interventions for these disorders. Second, more studies should be performed with longer follow-up durations, in order to provide more robust effect estimates and to establish long term effectiveness of psychological interventions. This is especially relevant as the risk of relapse persists over time [97]. Third, more research is needed on effective relapse prevention interventions combined with discontinuation of ADM, as most patients have reservations about the long-term use of medication [98], even though the risk of relapse during discontinuation is high [37]. Fourth, future studies should focus on determining the most effective components of relapse prevention interventions. As it might not be feasible to provide lengthy relapse prevention interventions to large groups of patients with remitted anxiety and depressive disorders, brief interventions using the most effective components could be a feasible alternative. This way, interventions might be more accessible and scalable and in turn have a wider impact.

## Conclusions

Psychological relapse prevention interventions are effective in reducing the risk of relapse for patients with remitted MDD, including over a long follow-up period of more than 3 years. Psychological relapse prevention interventions should be considered for all patients in remission from MDD. Due to a paucity of data, no conclusions could be drawn with regard to the effectiveness of relapse prevention interventions for anxiety disorders and the effectiveness of relapse prevention interventions combined with discontinuation of ADM. Future studies should focus on these topics.

## Supporting information

S1 Checklist PRISMA checklist. (DOC)

S1 File Search strategy. (DOCX)

S2 File Study characteristics. (DOCX)

S3 File Risk of bias appraisal. (DOCX)

S4 File Subgroup analyses. (DOCX)

S5 File Additional references. (DOCX)
