# Supplementary material for: Psychological interventions to prevent relapse in anxiety and depression: A systematic review and meta-analysis
Source: PLoS One. 2022 Aug 12;17(8):e0272200. doi: 10.1371/journal.pone.0272200 (PMC9374222; doi:10.1371/journal.pone.0272200)
Supplement: S1 File — (DOCX) [file pone.0272200.s002.docx]

# S1. Search strategy

The literature search was conducted in July 2021. Searches were performed in the following databases and article indexes (number of articles retrieved):

PubMed (2,551)

Embase (3,520)

PsycINFO (2.101)

To illustrate, the following Embase search is indicative of searches performed in other databases.

| **No.** | **Query** |
| --- | --- |
| #8 | #6 AND #7 |
| #7 | 'clinical trial'/de OR 'controlled clinical trial'/de OR 'randomized controlled trial'/de OR 'randomized controlled trial*':ab,ti,kw OR 'controlled clinical trial*':ab,ti,kw OR randomised:ab,ti,kw OR randomized:ab,ti,kw OR placebo*:ab,ti,kw OR randomly:ab,ti,kw OR rct:ab,ti,kw OR 'controlled trial*':ab,ti,kw OR 'clinical trial*':ab,ti,kw |
| #6 | #3 AND #4 AND #5 |
| #5 | 'relapse prevention'/exp OR 'remission'/exp OR 'recurrent disease'/exp OR 'relapse'/exp OR 'secondary prevention'/exp OR relaps*:ab,ti,kw OR remission*:ab,ti,kw OR recurren*:ab,ti,kw OR prevent*:ab,ti,kw OR exacerbat*:ab,ti,kw OR maintenan*:ab,ti,kw OR continuat*:ab,ti,kw OR discontinuat*:ab,ti,kw OR remitted*:ab,ti,kw |
| #4 | 'psychotherapy'/exp OR 'problem solving therapy'/exp OR psychotherap*:ab,ti,kw OR 'cognitive therap*':ab,ti,kw OR 'cognitive behaviour therap*':ab,ti,kw OR 'cognitive behavior therap*':ab,ti,kw OR 'cognitive behavioural therap*':ab,ti,kw OR 'cognitive behavioral therap*':ab,ti,kw OR mindfulnes*:ab,ti,kw OR mindfullnes*:ab,ti,kw OR 'problem solving*':ab,ti,kw OR 'psychodynamic therap*':ab,ti,kw OR 'psycho-dynamic therap*':ab,ti,kw OR 'psychoanalytic therap*':ab,ti,kw OR 'behaviour therap*':ab,ti,kw OR 'behavior therap*':ab,ti,kw OR 'behavioural therap*':ab,ti,kw OR 'behavioral therap*':ab,ti,kw OR 'psychological intervention*':ab,ti,kw OR 'psychological treatment*':ab,ti,kw OR 'psychological therap*':ab,ti,kw OR 'interpersonal therap*':ab,ti,kw OR (ipt:ab,ti,kw AND interperson*:ab,ti,kw) OR (pst:ab,ti,kw AND problem*:ab,ti,kw) OR 'implosive therap*':ab,ti,kw OR 'exposure therap*':ab,ti,kw OR 'maintenance cbt':ab,ti,kw OR 'maintenance ct':ab,ti,kw OR 'booster sessi*':ab,ti,kw OR 'solution focused therap*':ab,ti,kw |
| #3 | #1 OR #2 |
| #2 | 'anxiety disorder'/de OR 'generalized anxiety disorder'/exp OR 'panic'/exp OR 'phobia'/exp OR 'anxiety disorder*':ab,ti,kw OR agoraphobi*:ab,ti,kw OR 'panic disorder*':ab,ti,kw OR 'panic attack*':ab,ti,kw OR 'generalized anxiety disorder*':ab,ti,kw OR (gad:ab,ti,kw AND anxiet*:ab,ti,kw) OR 'generalised anxiety disorder*':ab,ti,kw OR claustrophobi*:ab,ti,kw OR phobi*:ab,ti,kw OR 'recurrent anxiet*':ab,ti,kw OR 'remitted anxiet*':ab,ti,kw OR ophidiophobi*:ab,ti,kw OR acrophobi*:ab,ti,kw |
| #1 | 'major depression'/exp OR 'major depression' OR 'chronic depression'/exp OR 'chronic depression' OR 'mixed anxiety and depression'/exp OR 'mixed anxiety and depression' OR 'recurrent brief depression'/exp OR 'recurrent brief depression' OR 'treatment resistant depression'/exp OR 'treatment resistant depression' OR 'major depression*':ab,ti,kw OR 'depressive disorder*':ab,ti,kw OR 'mood disorder*':ab,ti,kw OR 'severe depression*':ab,ti,kw OR mdd:ab,ti,kw OR 'recurrent depression*':ab,ti,kw OR 'remitted depression*':ab,ti,kw OR 'depressive episod*':ab,ti,kw OR 'chronic depression*':ab,ti,kw |
