# Supplementary material for: Psychological interventions to prevent relapse in anxiety and depression: A systematic review and meta-analysis
Source: PLoS One. 2022 Aug 12;17(8):e0272200. doi: 10.1371/journal.pone.0272200 (PMC9374222; doi:10.1371/journal.pone.0272200)
Supplement: S2 File — (DOCX) [file pone.0272200.s003.docx]

# S2 Study characteristics

Table S2 Study characteristics

| First author (year) | N | Type of disorder | Age | % female | Duration intervention in weeks (number of sessions) | Duration follow-up | Intervention  Group/Ind  Manualized: Y/N/? | Comparison | Setting | Definition of remission | Definition of relapse | Relapse rate intervention (%) | Relapse rate control (%) |
| --- | --- | --- | --- | --- | --- | --- | --- | --- | --- | --- | --- | --- | --- |
| Biesheuvel-Leliefeld et al. (2017) | 248 | MDD | 48.7 (11.7) | 69.8 | 8  (6) | 12 months | Self-help PCT  Ind.  Y | TAU | Community and Primary care | Full or partial remission of recurrent MDD | MDD according to SCID-I | 36 | 50 |
| Bockting et al. (2005) | 172 | MDD | 44.7 (9.5) | 73.5 | 8  (8) | 24 months | PCT + TAU  Group  Y | TAU | Community and Specialised care | Remission according to DSM–IV criteria, HRSD < 10 | MDD according to SCID-I | 56 | 64 |
| Bockting et al. (2009) | 172 | MDD | 44.7 (9.5) | 73.5 | 8  (8) | 66 months | PCT + TAU  Group  Y | TAU | Community and Specialised care | Remission according to DSM–IV criteria, HRSD < 10 | MDD according to SCID-I | 78 | 87 |
| Bockting et al. (2018) | 204 | MDD | 47.1 (9.9) | 66.5 | 8  (8) | 24 months | PCT + M-ADM  Ind or group  Y | M-ADM | Community, Primary and Specialised care | HRSD ≤ 10, last MDE ended at least 2 months and no longer than 2 years before study entry | Unclear | 43 | 60 |
| Bondolfi et al. (2010) | 60 | MDD | 47.5 | 71.6 | 8  (8 + 4 booster sessions) | 14 months | MBCT + TAU  Group  Y | TAU | Community, Primary and Specialised care | MADRS ≤13 | MDE according to SCID | 29 | 34 |
| de Jonge et al. (2019) | 214 | MDD | 43.4 (11.3) | 68.2 | 8  (8) | 15 months | PCT + TAU  Ind  Y | TAU | Specialised care | Remission according to SCID-I, HDRS <14 | MDE according to SCID-I | 23 | 33 |
| Fava et al. (1994) | 40 | MDD | 46.1 (3.7) | 67.5 | 20  (10) | 29 months | CBT + discontinuation ADM  Ind  N | Discontinuation ADM | Specialised care | Full remission & rated as ‘better’ or ‘much better’ on GRSI | RDC-defined MDE | 15 | 35 |
| Fava et al. (1998) | 40 | MDD | 46.9 (11.2) | 60 | 20  (10) | 29 months | CBT + discontinuation ADM  Ind  N | Discontinuation ADM | Specialised care | Full remission & rated as ‘better’ or ‘much better’ on GRSI | RDC-defined MDE | 25 | 80 |
| Frank (1990) | 53 | MDD | 40.2 (10.9) | 76.6 | 156  (36) | 36 months | M-IPT + M-ADM  Ind  N | M-ADM | Unknown | HRSD ≤ 7& RSD ≤ 5 for a total of 20 weeks | RDC-defined MDD AND HRSD ≥ 15 AND RSD ≥ 7 | 24 | 21 |
| Godfrin and Van Heeringen (2010) | 106 | MDD | 45.7 (10.6) | 81.1 | 8  (8) | 14 months | MBCT + TAU  Group  Y | TAU | Specialised care | HRSD <14, no current MDE according to DSM-IV & end of the last episode being at least 8 weeks before study entry | MDD according to SCID-I | 30 | 68 |
| Holländare et al. (2011) | 84 | MDD | 45.3 (12.8) | 84.5 | 10  (10) | 8 months | Internet-based CBT  Ind  Y | TAU | Community | BDI ≤ 10 | MDD according to DSM-IV | 11 | 38 |
| Holländare et al. (2013) | 67 | MDD | 45.3 (12.8) | 84.5 | 10  (16) | 26 months | Internet-based CBT  Ind  Y | TAU | Community | BDI ≤ 10 | MDD according to DSM-IV | 14 | 61 |
| Huijbers et al. (2015) | 68 | MDD | 51.8 (14.2) | 72 | 8  (8) | 15 months | MBCT + M-ADM  Group  Y | M-ADM | Specialised care | Full (≤11 on IDS-C) or partial (>11 on IDS-C) remission, not meeting DSM-IV criteria for MDD | MDE according to SCID-I | 36 | 37 |
| Jarrett et al. (2000) | 14 | MDD | 41.2 (10.1) | 84 | 35  (10) | 24 months | C-CT  Ind  N | TAU | Specialised care | HRSD ≤ 9, no MDD | RDC-defined MDD OR patient had treatment | 40 | 83 |
| Jarrett et al. (2001) | 84 | MDD | 42.7 (1.14) | 72.6 | 35  (10) | 24 months | C-CT  Ind  Y | TAU | Specialised care | HRSD-17 ≤ 9, no MDD | MDE according to DSM-IV criteria, using LIFE | 39 | 50 |
| Klein et al. (2004) | 82 | MDD | 45.1 (11.4) | 67.1 | 52  (13) | 12 months | CBASP  Ind  N | TAU | Specialised care | Reduction from the acute phase baseline of at least 50% and HRSD-24 ≤ 15 | HRSD-24 ≥ 16 on two consecutive visits AND MDD according to DSM-IV | 3 | 21 |
| Klein et al. (2018) | 264 | MDD | 46 (10.8) | 75 | 13  (8) | 24 months | Online mobile- CT  Ind  Y | TAU | Community, Primary and Specialised care | HRSD ≤ 10, in remission for at least 8 weeks but no longer than 24 months according to SCID-I | MDD according to SCID | 44 | 49 |
| Ma and Teasdale (2004) | 75 | MDD | 44.5 (9.0) | 76 | 34  (8) | 14 months | MBCT  Group  Y | TAU | Community and Primary care | HAMD <10 | MDE according to SCID | 39 | 62 |
| Meadows et al. (2014) | 203 | MDD | 48.4 (12.4) | 81.3 | 8  (8) | 26 months | MBCT + TAU  Group  Y | TAU | Primary and Specialised care | No current MDE | Unclear | 45 | 55 |
| Morokuma et al. (2013) | 34 | MDD | 42.8 | 56.2 | 6  (6) | 10 months | PE + TAU  Group  Y | TAU | Specialised care | Remission: HRSD ≤ 6, partial remission: according to DSM- IV | MDE according to DSM-IV | 6 | 36 |
| Paykel et al. (1999) | 158 | MDD | 43.4 (10.5) | 49.5 | 20  (18) | 16 months | CT + M-ADM  Ind  Y | M-ADM | Specialised care | HDRS <8, BDI <9 at 2 successive ratings 4 weeks apart | MDD according to DSM-III-R for a minimum of 1 month AND at 2 successive face-to-face assessments required to meet severity criteria for MDD AND HDRS ≥ 17 | 23 | 34 |
| Paykel et al. (2005) | 158 | MDD | 43.4 (10.5) | 49.5 | 20  (18) | 63 months | CT + M-ADM  Ind  Y | M-ADM | Specialised care | HDRS <8, BDI <9 at 2 successive ratings 4 weeks apart | MDD according to DSM-III-R for a minimum of 1 month AND at 2 successive face-to-face assessments required to meet severity criteria for MDD AND HDRS ≥ 17 | 60 | 65 |
| Perlis et al. (2002) | 132 | MDD | 39.9 (10.3) | 54.5 | 26  (19) | 6 months | CBT + M-ADM  Ind  Y | M-ADM | Specialised care | HAMD-17 ≤ 7 for at least 3 weeks | MDE according to DSM OR HAMD-17 ≥ 15 at two consecutive visits | 6 | 8 |
| Petersen et al. (2010) | 26 | MDD | 43.6 | 54 | 80  (23) | 20 months | CBT + M-ADM  Ind  Y | M-ADM | Specialised care | HAMD-17 ≤7 for 3 consecutive weeks | MDD according to SCID OR HAMD-17 ≥ 15 at two consecutive visits | 36 | 29 |
| Petersen et al. (2010) | 29 | MDD | 43.6 | 54 | 80  (23) | 20 months | CBT + placebo  Ind  Y | Placebo | Specialised care | HAMD-17 ≤7 for 3 consecutive weeks | MDD according to SCID OR HAM-D-17 ≥ 15 at two consecutive visits | 45 | 50 |
| Reynolds et al. (1999) | 53 | MDD | 67.6 (5.8) | 74.9 | 156  (36) | 36 months | IPT + M-ADM  Ind  Y | M-ADM | Specialised care | HRSD-17 ≤ 10 for 3 consecutive weeks | RDC-defined MDE | 20 | 43 |
| Reynolds et al. (1999) | 54 | MDD | 67.6 (5.8) | 74.9 | 156  (36) | 36 months | IPT + placebo  Ind  Y | Placebo | Specialised care | HRSD-17 ≤ 10 for 3 consecutive weeks | RDC-defined MDE | 64 | 90 |
| Reynolds et al. (2006) | 63 | MDD | 77.3 (6.4) | 63.6 | 104  (24) | 24 months | IPT + M-ADM  Ind  Y | M-ADM | Specialised care | HRSD ≤10 | MDE according to DSM-IV AND HRSD ≥ 15 | 29 | 34 |
| Reynolds et al. (2006) | 53 | MDD | 76.5 (4.9) | 65.9 | 104  (24) | 24 months | IPT + placebo  Ind  Y | Placebo | Specialised care | HRSD ≤10 | MDE according to DSM-IV AND HRSD ≥ 15 | 60 | 56 |
| Scholten et al. (2018) | 87 | Anxiety disorder | 41.7 (12.7) | 60 | 17  (8) | 16 months | CBT + discontinuation ADM  Group  Y | Discontinuation ADM | Community, Primary and Specialised care | No disorder, according to SCID-I | Anxiety disorder OR MDD according to SCID-I | 61 | 58 |
| Segal et al. (2010) | 56 | MDD | 44 (11) | 63 | 8  (8) | 18 months | MBCT + discontinuation ADM  Group  Y | Discontinuation ADM | Community, Primary and Specialised care | Stable remission: HRSD ≤ 7, unstable remission HRSD ≤ 7 with occasional elevation to 8-14 | MDD according to SCID-I, for at least 2 weeks | 38 | 60 |
| Segal et al. (2020) | 460 | MDD | 48.3 (14.9) | 75.6 | 12  (8) | 15 months | Online-MBCT  Ind  Y | TAU | Primary care | PHQ-9 ≥5 and ≤9, residual depressive symptoms | PHQ-9 ≥15 | 14 | 23 |
| Shallcross et al. (2015) | 92 | MDD | 34.9 (11.4) | 76 | 8  (8) | 14 months | MBCT  Group  Y | TAU | Community and Primary care | Remission of at least 1 month prior to interview | MDD according to SCID | 33 | 30 |
| Shallcross et al. (2018) | 92 | MDD | 34.9 (11.4) | 76 | 8  (8) | 26 months | MBCT  Group  Y | TAU | Community and Primary care | Remission of at least 1 month prior to interview | MDD according to SCID | 48 | 50 |
| Stangier et al. (2013) | 180 | MDD | 48.6 (11.6) | 72.2 | 35  (16) | 20 months | M-CBT  Ind  Y | TAU | Community and Specialised care | HAM-D ≤9 in 8 weeks before randomisation | MDE according to DSM-IV criteria, using LIFE | 51 | 60 |
| Teasdale et al. (2000) | 145 | MDD | 41.3 (10.6) | 76 | 8  (8) | 14 months | MBCT  Group  Y | TAU | Community | Remission or recovery, HRSD < 10 | MDE according to SCID | 44 | 58 |
| White et al. (2013) | 157 | Anxiety disorder | 37.8 (11.9) | 66.8 | 39  (9) | 21 months | M-CBT  Ind  Y | TAU | Specialised care | 40% reduction of PDSS-IE score relative to baseline & CGI score of ‘much’ or ‘very much’ improved relative to baseline study entry | 2 weeks with 1) ≥ 40% increase in PDSS score relative to post-acute treatment score and 2) CGI score of ‘much worse’ or ‘very much worse’ relative to post-acute treatment status | 5 | 18 |
| Wilkinson et al. (2009) | 45 | MDD | 74.0 (7.3) | 62.2 | 10  (8) | 12 months | CBT  Group  Y | TAU | Primary and Specialised care | MADRS <10, remitted for at least 2 months | MADRS ≥10 | 28 | 44 |
| Williams et al. (2014) | 136 | MDD | 43 (12) | 72 | 8  (8) | 12 months | MBCT  Group  Y | TAU | Community, Primary and Specialised care | Remission for the previous 8 weeks (no core symptom of depression or suicidal feelings during at least 1 week) | MDD according to SCID criteria, for at least 2 weeks | 46 | 53 |
| Williams et al. (2014) | 138 | MDD | 43 (12) | 72 | 8  (8) | 12 months | CPE  Group  Y | TAU | Community, Primary and Specialised care | Remission for the previous 8 weeks (no core symptom of depression or suicidal feelings during at least 1 week | MDD according to SCID criteria, for at least 2 weeks | 50 | 53 |

**Abbreviations**

Interventions:

PCT = Preventive Cognitive Therapy, CBT = Cognitive Behavioural Therapy, TAU = Treatment As Usual, MBCT = Mindfulness-Based Cognitive Therapy, M-IPT = Maintenance Interpersonal Psychotherapy, IPT = Interpersonal Psychotherapy, C-CT = Continuation-Cognitive Therapy, M-ADM = Maintenance Antidepressant Medication, CBASP = Cognitive Behavioural Analysis System of Psychotherapy, (C)PE = (Cognitive) Psychoeducation, CT = Cognitive Therapy, M-CBT = Maintenance-Cognitive Behavioural Therapy

Measures:

BDI = Beck Depression Inventory, SCID = Structured Clinical Interview for DSM-IV, HRSD/HDRS/HAMD = Hamilton Rating Scale for Depression, MADRS = Montgomery-Åsberg Depression Rating Scale, RDC = Research Diagnostic Criteria, GRSI = Global Rating Scale of Improvement, RSD = Raskin Severity of Depression, LIFE = Longitudinal Interval Follow-up Evaluation, CGI = Clinical Global Improvement

Other:

MDD = Major Depressive Disorder, MDE = Major Depressive Episode
