# Supplementary material for: Psychological interventions to prevent relapse in anxiety and depression: A systematic review and meta-analysis
Source: PLoS One. 2022 Aug 12;17(8):e0272200. doi: 10.1371/journal.pone.0272200 (PMC9374222; doi:10.1371/journal.pone.0272200)
Supplement: S3 File — (DOCX) [file pone.0272200.s004.docx]

# S3 Risk of bias appraisal

Table S3 Risk of bias appraisal

| First author | Year | Random sequence generation | Allocation concealment | Blinding of outcome assessment | Incomplete outcome data | Selective reporting | Other bias |
| --- | --- | --- | --- | --- | --- | --- | --- |
| Biesheuvel-Leliefeld | 2017 | Low risk | High risk | Low risk | Low risk | High risk | Low risk |
| Bockting | 2005 | Low risk | Low risk | Low risk | Low risk | Unclear | Low risk |
| Bockting_FU | 2009 | Low risk | Low risk | Low risk | Unclear | Unclear | Low risk |
| Bockting | 2018 | Low risk | Low risk | Low risk | Unclear | Low risk | Low risk |
| Bondolfi | 2010 | Low risk | Low risk | Low risk | Low risk | Unclear | Low risk |
| de Jonge | 2019 | Low risk | Low risk | Low risk | Low risk | High risk | Unclear |
| Fava | 1994 | Unclear | Unclear | Low risk | Low risk | Unclear | High risk |
| Fava | 1998 | Unclear | Unclear | Unclear | Low risk | Unclear | Unclear |
| Frank | 1990 | Unclear | Unclear | Low risk | Low risk | Unclear | Low risk |
| Godfrin | 2010 | Low risk | Low risk | Unclear | Low risk | Unclear | Unclear |
| Holländare | 2011 | Low risk | Unclear | Unclear | Low risk | Unclear | Low risk |
| Holländare_FU | 2013 | Low risk | Unclear | Unclear | Low risk | Unclear | Low risk |
| Huijbers | 2015 | Low risk | Low risk | High risk | High risk | Low risk | High risk |
| Jarrett | 2000 | Unclear | Unclear | High risk | Unclear | Unclear | High risk |
| Jarrett | 2001 | Low risk | Unclear | High risk | Low risk | Unclear | Low risk |
| Klein | 2004 | Unclear | Unclear | Low risk | Low risk | Unclear | High risk |
| Klein | 2018 | Low risk | Low risk | Low risk | Low risk | Low risk | Low risk |
| Ma | 2004 | Unclear | High risk | Low risk | Unclear | Unclear | Unclear |
| Meadows | 2014 | Low risk | Low risk | Low risk | Low risk | High risk | Low risk |
| Morokuma | 2013 | Low risk | Low risk | Low risk | Low risk | Unclear | High risk |
| Paykel | 1999 | Low risk | Low risk | Low risk | Low risk | Unclear | Unclear |
| Paykel_FU | 2005 | Low risk | Low risk | Low risk | Low risk | Unclear | Unclear |
| Perlis | 2002 | Unclear | Unclear | Low risk | High risk | Unclear | Unclear |
| Petersen | 2010 | Unclear | Unclear | Low risk | Low risk | Unclear | High risk |
| Reynolds | 2006 | Low risk | Low risk | Low risk | Unclear | High risk | Low risk |
| Reynolds | 1999 | Low risk | Low risk | Low risk | Low risk | Unclear | Low risk |
| Scholten | 2018 | Low risk | Low risk | High risk | Low risk | Low risk | High risk |
| Segal | 2010 | Low risk | Low risk | Low risk | Low risk | Unclear | Unclear |
| Segal | 2020 | Low risk | Low risk | Low risk | Unclear | Low risk | High risk |
| Shallcross | 2015 | Low risk | Low risk | Low risk | High risk | Unclear | Unclear |
| Shallcross_FU | 2018 | Low risk | Low risk | Low risk | High risk | Unclear | Unclear |
| Stangier | 2013 | Low risk | Low risk | Low risk | High risk | Low risk | Low risk |
| Teasdale | 2000 | Low risk | Low risk | Low risk | Low risk | Unclear | Low risk |
| White | 2013 | Unclear | Unclear | Low risk | Low risk | Unclear | High risk |
| Wilkinson | 2009 | Low risk | Low risk | Low risk | Low risk | Unclear | Low risk |
| Williams | 2014 | Low risk | Low risk | Low risk | Unclear | Low risk | Low risk |
