# Supplementary material for: Psychological interventions to prevent relapse in anxiety and depression: A systematic review and meta-analysis
Source: PLoS One. 2022 Aug 12;17(8):e0272200. doi: 10.1371/journal.pone.0272200 (PMC9374222; doi:10.1371/journal.pone.0272200)
Supplement: S4 File — (DOCX) [file pone.0272200.s005.docx]

# S4 Subgroup analyses

Fig. S4.1 Subgroup analysis on whether patients had received an intervention (psychological or pharmacological) prior to the relapse prevention intervention

Fig. S4.2 Subgroup analysis on type of intervention
