# Supplementary material for: Psychological interventions to prevent relapse in anxiety and depression: A systematic review and meta-analysis
Source: PLoS One. 2022 Aug 12;17(8):e0272200. doi: 10.1371/journal.pone.0272200 (PMC9374222; doi:10.1371/journal.pone.0272200)
Supplement: S5 File — (DOCX) [file pone.0272200.s006.docx]

# S5 References of articles solely included in meta-analysis

1. Biesheuvel-Leliefeld KEM, Dijkstra-Kersten SMA, van Schaik DJF, van Marwijk HWJ, Smit F, van der Horst HE, et al. Effectiveness of Supported Self-Help in Recurrent Depression: A Randomized Controlled Trial in Primary Care. Psychother Psychosom [Internet]. 2017;86(4):220–30. Available from: https://www.karger.com/Article/FullText/472260

2. Bockting CLH, Klein NS, Elgersma HJ, van Rijsbergen GD, Slofstra C, Ormel J, et al. Effectiveness of preventive cognitive therapy while tapering antidepressants versus maintenance antidepressant treatment versus their combination in prevention of depressive relapse or recurrence (DRD study): a three-group, multicentre, randomised control. The Lancet Psychiatry [Internet]. 2018 May;5(5):401–10. Available from: http://dx.doi.org/10.1016/S2215-0366(18)30100-7

3. Frank E. Three-Year Outcomes for Maintenance Therapies in Recurrent Depression. Arch Gen Psychiatry [Internet]. 1990 Dec 1;47(12):1093. Available from: http://archpsyc.jamanetwork.com/article.aspx?doi=10.1001/archpsyc.1990.01810240013002

4. Huijbers MJ, Spinhoven P, Spijker J, Ruhé HG, Van Schaik DJF, Van Oppen P, et al. Adding mindfulness-based cognitive therapy to maintenance antidepressant medication for prevention of relapse/recurrence in major depressive disorder: Randomised controlled trial. J Affect Disord [Internet]. 2015;187:54–61. Available from: http://dx.doi.org/10.1016/j.jad.2015.08.023

5. Jarrett RB, Kraft D, Doyle J, Foster BM, Eaves GG, Silver PC. Preventing Recurrent Depression Using Cognitive Therapy With and Without a Continuation Phase. Arch Gen Psychiatry [Internet]. 2001 Apr 1;58(4):381. Available from: http://archpsyc.jamanetwork.com/article.aspx?doi=10.1001/archpsyc.58.4.381

6. Jarrett RB, Kraft D, Schaffer M, Witt-Browder A, Risser R, Atkins DH, et al. Reducing Relapse in Depressed Outpatients with Atypical Features: A Pilot Study. Psychother Psychosom [Internet]. 2000;69(5):232–9. Available from: https://www.karger.com/Article/FullText/12401

7. Klein NS, Kok GD, Burger H, van Valen E, Riper H, Cuijpers P, et al. No Sustainable Effects of an Internet-Based Relapse Prevention Program over 24 Months in Recurrent Depression: Primary Outcomes of a Randomized Controlled Trial. Psychother Psychosom [Internet]. 2018;87(1):55–7. Available from: http://www.embase.com/search/results?subaction=viewrecord&from=export&id=L624445633 U2 - L624445633

8. Meadows GN, Shawyer F, Enticott JC, Graham AL, Judd F, Martin PR, et al. Mindfulness-based cognitive therapy for recurrent depression: A translational research study with 2-year follow-up. Aust New Zeal J Psychiatry [Internet]. 2014 Aug 4;48(8):743–55. Available from: http://journals.sagepub.com/doi/10.1177/0004867414525841

9. Morokuma I, Shimodera S, Fujita H, Hashizume H, Kamimura N, Kawamura A, et al. Psychoeducation for major depressive disorders: A randomised controlled trial. Psychiatry Res [Internet]. 2013;210(1):134–9. Available from: http://dx.doi.org/10.1016/j.psychres.2013.05.018

10. Stangier U, Hilling C, Heidenreich T, Risch AK, Barocka A, Schlösser R, et al. Maintenance Cognitive-Behavioral Therapy and Manualized Psychoeducation in the Treatment of Recurrent Depression: A Multicenter Prospective Randomized Controlled Trial. Am J Psychiatry [Internet]. 2013 Jun;170(6):624–32. Available from: http://psychiatryonline.org/doi/abs/10.1176/appi.ajp.2013.12060734

11. Teasdale JD, Segal Z V., Williams JMG, Ridgeway VA, Soulsby JM, Lau MA. Prevention of relapse/recurrence in major depression by mindfulness-based cognitive therapy. J Consult Clin Psychol [Internet]. 2000;68(4):615–23. Available from: http://doi.apa.org/getdoi.cfm?doi=10.1037/0022-006X.68.4.615

12. Wilkinson P, Alder N, Juszczak E, Matthews H, Merritt C, Montgomery H, et al. A pilot randomised controlled trial of a brief cognitive behavioural group intervention to reduce recurrence rates in late life depression. Int J Geriatr Psychiatry [Internet]. 2009 Jan;24(1):68–75. Available from: http://doi.wiley.com/10.1002/gps.2076
